# Herbal Simples Approved for Modern Uses of Cure

**Published:** 1896-03

**Authors:** 


**Herbal Simples approved for Modern Uses of Cure.** By W. T. Fernie, M.D. Pp. xvi., 432. Bristol: John Wright & Co. 1895.

Dr. Fernie has brought together in this volume a large mass of information concerning vegetable remedies in use now or in past days. His researches into old Herbals and such like books have been extensive, and very interesting is the result of his labours. Not contented with solely giving the virtues which each plant is reputed by tradition to possess, he also endeavours to show the active principles upon which the pharmacological and therapeutical properties of each remedy depend. At times he would seem to draw to some degree upon his readers' credulity. For example, he says that the Sundew "given experimentally to cats has been found to stud the surface of their lungs with true tubercular matter. . . . Meantime it has been well demonstrated (by Dr. Curie, and others) that at the onset of pulmonary consumption in the human subject a cure may nearly always be brought about, or the symptoms materially improved, by giving the tincture of *Sundew*." The substances treated of are arranged alphabetically, so that the brief therapeutical index provided is all-sufficient.

